# Influence of negative affectivity and self-esteem on the oral health related quality of life in patients receiving oral rehabilitation

**DOI:** 10.1186/1477-7525-11-178

**Published:** 2013-10-24

**Authors:** Esben Boeskov Özhayat

**Affiliations:** 1 Section of Oral Rehabilitation, Department of Odontology, Faculty of Health Science, University of Copenhagen, Nørre Allé 20, Copenhagen DK-2200, Denmark

**Keywords:** Personality, Quality of life, Prosthodontics, Treatment outcome, Data interpretation

## Abstract

**Background:**

The aim of this study was to investigate if and how the personality traits Negative Affectivity (NA) and self-esteem influenced the Oral Health Related Quality of Life (OHRQoL) in patients receiving oral rehabilitation.

**Methods:**

OHRQoL was measured by the Oral Health Impact Profile 49 (OHIP-49), NA with a short form of the Eysenck Personality Inventory Questionnaire (EPI-Q), and self-esteem with Rosenbergs Self-Esteem Scale (RSES) in 66 patients treated with removable dental prosthesis (RDP). The minimally important difference (MID), effect size (ES), and standard error of the measurement (SEM) were used to clinically interpret the patient-reported effect.

**Results:**

The OHIP-49 score was significantly higher and exceeded the MID pre- and post-treatment in participants with high EPI-Q and low RSES score compared to participants with low EPI-Q and high RSES score. The improvement in OHIP-49 score was significant and not limited by high EPI-Q and low RSES score. High EPI-Q score was associated high improvement in OHIP-49 score and the ES of the improvement in participants with high EPI-Q was large and exceeded the MID and SEM.

**Conclusion:**

Treatment with RDP improves the OHRQoL regardless of level of NA and self-esteem. High NA is associated with a large effect, but both high NA and low self-esteem is associated with poorer OHRQoL both before and after treatment.

## Introduction

Tooth loss is a common problem which can lead to different functional limitations and a reduction in quality of life [1]. The impact of the oral condition on quality of life, the Oral Health Related Quality of Life (OHRQoL) is therefore crucial to evaluate when oral rehabilitation of patients with tooth loss is intended and also to determining if the rehabilitation had the intended patient-reported effect [2]. When rehabilitating patients with partial tooth loss, removable dental prostheses (RDP) are still a widely used treatment option, and studies have shown that the treatment often leads to an improvement in OHRQoL [3,4]. It has, however, also been shown that the OHRQoL deteriorates in some patients treated with RDP and that an unfortunate high risk exist that an RDP will not be used when provided [3,5,6]. Therefore, the clinician should consider all factors that could influence the effect of treatment, and even though different factors have been proposed to influence the use and OHRQoL in these patients [5-7], it is not fully understood in which cases the treatment has the intended effect and in which cases it does not.

Personality is a factor not investigated in relation to patient-reported effect of an RDP but said to be important when interpreting OHRQoL measures [8]. A deeper understanding of the influence of personality traits is necessary if clinical investigations using OHRQoL measures are to be meaningfully interpreted [9], and especially interesting if OHRQoL measures are used to guide clinical decisions and evaluate treatment outcome [10,11]. Negative affectivity (NA) is a personality trait that leads to the experience of subjective distress, including aversive mood states such as anger, disgust, scorn, guilt, fearfulness and depression [12]. NA is considered important to quality of life [13], and can been measured by different questionnaires [12]. A shortened version of the Eysenck Personality Inventory Questionnaire (EPI-Q) [14] was in a population study relating NA to OHRQoL used to show that high NA was associated with poor OHRQoL [10]. The influence of NA on the patient-reported effect of treatment with RDP in partially toothless patients has not been studied but would be highly relevant to investigate, as it could be hypothesized that high NA would be associated with less patient-reported effect of treatment. Another personality trait important to quality of life is self-esteem [9]. In medicine, it has been reported that self-esteem is an important factor to consider when predicting the outcome of health treatments [15-17], and self-esteem has been said to be captured by OHRQoL measures [18]. Self-esteem would therefore also be a relevant personality trait to include in a study on patient-reported effect of treatment with RDP.

Few prospective intervention studies relating NA or self-esteem with health related quality of life have been published [19,20] and none are concerned with oral health. In an earlier study, it was found that low self-esteem and high NA was associated with worse OHRQoL in patients with partial tooth loss before receiving an RDP [21]. This study is a follow-up on the baseline findings and investigates if and how the personality traits NA and self-esteem influence the OHRQoL in patients receiving oral rehabilitation with RDP. The hypothesis was that high NA and low self-esteem would be associated with poorer effect of treatment as measured by OHRQoL.

## Material and methods

### Participants

Eighty-one patients with partial tooth loss attending the clinic at the Section of Oral Rehabilitation, Department of Odontology, University of Copenhagen between September 2008 and September 2011 for treatment with RDP completed the pre-treatment questionnaires and had an oral examination and history taking done. The patients were included consecutively and the treating student and the clinical instructor collected the questionnaires from the patient both before and after treatment. The follow-up was conducted 1 month after finalizing treatment and the length between baseline and follow-up measurement was between 5 and 10 month depending on the length of treatment. If a patient presented with acute pain, profound caries lesions, periodontal treatment need not manageable by motivation, instruction and light cleaning, and/or need for temporomandibular joint treatment, the patient was excluded. The participants should further be capable of reading and answering the questionnaires in Danish. The post-treatment questionnaires were completed by 66 (34 women and 32 men, age range 38–83) of the 81 participants (81.5%).

### OHRQoL

OHRQoL was measured before and after treatment by a Danish version of the Oral Health Impact Profile 49 (OHIP-49) questionnaire [22]. The OHIP-49 consisted of 49 questions related to problems in the oral region [23], and the participants answered how often each problem had occurred during the past month on a scale with six choices and according scores: very often (4), *fairly often* (3), *occasionally* (2), *hardly ever* (1), *never* (0) or *don’t know*. The scores from the 49 answers were added, giving an OHIP-49 score between 0 and 196. A non-adjusted pre-treatment score and a pre-treatment score adjusted for the regression to the mean (RTM) were calculated. The adjustment ensured that the statistical tendency of extreme scores to become less extreme at follow-up was minimized [24]. The change in OHIP-49 score was calculated by subtracting the pre-treatment scores from the post-treatment score.

### Negative affectivity

NA was measured pre-treatment by the short form of the Eysenck Personality Inventory Questionnaire (EPI-Q) [14], which consisted of 9 questions regarding affection with dichotomous answers (yes/no). A point was given each time a question was answered “yes” giving a score for each participant between 0 and 9 and a higher score indicating higher NA. To distinguish between participants with high and low NA, the participants were allocated to a high EPI-Q group and a low EPI-Q group based on the mean EPI-Q score in the participants. Participants with a score of 4–9 were allocated to the high EPI-Q group and participants with a score of 0–3 were allocated to the low EPI-Q group.

### Self-esteem

Self-esteem was measured pre-treatment by Rosenbergs Self-Esteem Scale (RSES) [25], which consisted of 10 items regarding self-esteem. Each item was rated on a 4-point response scale, 1 being “strongly agree” and 4 “strongly disagree”. Addition of the item scores gave an overall score from 10–40 with higher score indicating higher self-esteem. To distinguish between participants with high and low self-esteem, the participants were allocated to a high RSES group and a low RSES group based on the mean RSES score. Participants with a score of 31–40 were allocated to the high RSES group and participants with a score of 30 or below were allocated to the low RSES group. This division is in accordance with other studies dividing the RSES score into high and low self-esteem groups [26-28].

### Controlling variables

Controlling variables were obtained from the history takings and oral examinations performed by students approved by a clinical teacher using a written guideline used at the Section. The controlling variables included in the study were: gender, age, number of teeth replaced, experience of wearing RDP (yes/no), location of replaced teeth (one jaw or both), and zone of replaced teeth (masticatory or masticatory/aesthetic). The aesthetic zone was defined as incisors, canines and 1^st^ premolars in the upper jaw and incisors and canines in the lower. The masticatory zone was defined as the 2^nd^ premolars and the 1^st^ and 2^nd^ molars in the upper jaw and both premolars and 1^st^ and 2^nd^ molars in the lower jaw [29].

### Analyses

SAS® (version 9.2, SAS Institute Inc., Cary, NC, USA) and SPSS (version 19.0, SPSS inc. IBM® Company) statistical software was used for the analyses. The statistical significance level was P < 0.05.

Descriptive statistics was used to calculate means and frequency distributions. Correlations between OHIP-49 scores and EPI-Q score, and RSES score were performed using Pearson’s correlation. The linear relationship between the OHIP-49 change scores and the EPI-Q and RSES scores was tested by linear regression analyses. Student’s t-test was used to test differences in OHIP-49 scores between the EPI-Q and RSES groups and paired t-tests were used to test differences within the groups.

Adjustment for RTM was done using the Edwards-Nunnally method [30]:

$$\begin{array}{l} \mathrm{Adjust}\;\mathrm{score}=r_{\mathrm{xx}}\left(X_{A}-M\right)+M\\ \; \end{array}$$

Where *r*_*xx*_ is the test-retest reliability, *X*_*A*_ is the raw admission score, and *M* is the mean admission score. Based on the test-retest reliability found in other studies *r*_*xx*_ was set to 0.88 [31-33].

Multivariate models were created to test how much of the variance in adjusted OHIP-49 change scores was attributed to the EPI-Q and RSES score. The first model included the controlling variables only, EPI-Q score was added in the second model, RSES score in the third model, and both EPI-Q and RSES score in the fourth. Parameter estimates of the RSES and EPI-Q scores were derived from the fourth model to investigate to what extent the scores explained the change in OHIP-49 score. To investigate the difference between participants with high and low self-esteem and NA respectively, a fifth model was created including the controlling variables and the RSES and EPI-Q groups. Parameter estimates of the groups were derived from the model.

The drop-out analyses showed no difference between drop-outs and participants in mean age, number of teeth and OHIP-49, EPI-Q and RSES score as tested by t-tests. The frequency of EPI-Q group, RSES group, gender, experience of RDP, and zone and location of missing teeth between drop-outs and participants was also not significantly different as tested by chi-square tests (Table 1).

**Table 1:** Mean scores and distribution

	**Baseline (n = 81)**	**Follow-up (n = 66)**
**Means**		
**OHIP-49 score (SD)**		
Before mean non-adjusted	50.8 (35.5)	49.00 (35.4)
EPI-Q group		
Low	37.4 (29.5)	35.1 (30.8)
High	67.7 (35.5)*	65.6 (33.8)*
RSES group		
Low	62.0 (39.9)*	62.8 (37.8)*
High	44.6 (31.5)	40.6 (31.5)
Before mean adjusted	50.8 (31.2)	49.04 (31.2)
EPI-Q group		
Low	39.0 (26.0)	36.8 (27.6)
High	65.6 (31.2)*	63.7 (26.8)*
RSES group		
Low	60.7 (35.2)*	61.1 (33.2)*
High	45.4 (27.7)	41.7 (27.7)
After mean	-	30.41 (30.1)
EPI-Q group		
Low	-	23.4 (25.5)
High	-	38.8 (34.2)*
RSES group		
Low	-	41.0 (40.4)*
High	-	24.0 (19.5)
**EPI-Q score**		
Mean (SD)	3.2 (2.4)	3.4 (2.6)
**RSES score**		
Mean (SD)	32.3 (5.9)	31.9 (6.3)
**Age**		
Mean (SD)	63.7 (11.7)	64.8 (10.2)
**Number of teeth replaced**		
Mean (SD)	-	7.6 (3.6)
		
**Distribution of participants**		
**EPI-Q group**		
High	36 (44%)	30 (45%)
Low	45 (56%)	36 (55%)
**RSES group**		
High	52 (64%)	41 (62%)
Low	29 (36%)	25 (38%)
**Gender**		
Women	43 (53%)	34 (52%)
Men	38 (47%)	32 (48%)
**Experience RDP**		
Yes	49 (60%)	41 (62%)
No	32 (40%)	25 (38%)
**Location of missing teeth**		
One jaw	64 (80%)	55 (83%)
Both	17 (20%)	11 (17%)
**Zone of missing teeth**		
Masticatory	22 (27%)	17 (26%)
Masticatory/aesthetic	59 (73%)	49 (74%)

*Denotes significant difference between high and low EPI-Q and RSES group respectively (P < 0.05).

To test if the statistically significant differences between pre-treatment and post-treatment OHIP-49 scores were clinically meaningful, the distribution-based methods effect size (ES) [34] and standard error of the measurement (SEM) [35] was calculated within each group of participants. An effect of 0.2 was considered small, 0.5 moderate, and 0.8 large [34] and if the change in OHIP-49 score within a subgroup exceeded the SEM, the difference was considered clinically significant [36].

To evaluate the clinical meaning of the differences in OHIP-49 score between and within the high and low EPI-Q and RSES groups, the upper limit of the minimally important difference (MID) found by John et al. [37] to be 9 OHIP-49 units was applied.

## Results

### Means and distribution

The means of the OHIP-49, EPI-Q and RSES scores as well as the distribution of the participants according to EPI-Q group, RSES group, and controlling variables are presented in Table 1. It is seen that the OHIP-49 score was significantly higher both pre- and post-treatment in the high EPI-Q and low RSES group compared to the low EPI-Q and high RSES group.

### Bivariate analyses of OHIP-49 scores and clinical meaning

Before treatment a significant correlation between the OHIP-49 score and the RSES score (R = -0.34) and the EPI-Q score (R = 0.48) was found for the entire population. The EPI-Q score only was significantly correlated to the post-treatment OHIP-49 score (R = 0.42).

The association between the OHIP-49 change scores and the EPI-Q score showed a non-significant correlation and linear relationship with R = -0.16 and β-coefficient = -4.4 (P = 0.21) for the non-adjusted score and R = -0.09 and β-coefficient = -3.4 (P = 0.47) for the adjusted score. The association between the OHIP-49 change scores and the RSES score showed a significant correlation and linear relationship with R = 0.27 and β-coefficient = 0.12 (P = 0.03) for the non-adjusted scores and a non-significant correlation and linear relationship with R = 0.23 and β-coefficient = -0.03 (P = 0.06) for the adjusted score.

Non-adjusted and adjusted OHIP-49 change scores, effect sizes, and SEM for the EPI-Q and RSES groups are shown in Table 2. All groups decreased significantly in OHIP-49 score after treatment. The high EPI-Q and RSES group presented large effect after adjustment and also showed clinical significant change as determined by the SEM. When applying the MID, all groups improved more than 9 OHIP-49 units. A significant higher improvement in OHIP-49 score was seen in the high EPI-Q group compared to the low EPI-Q group before but not after adjustment of the OHIP-49 score.

**Table 2:** Change in mean OHIP-49 scores (CI) and according ES and SEM

	**Non-adjusted**			**Adjusted**		
	**Mean score**	**ES**	**SEM**	**Mean score**	**ES**	**SEM**
**All participants**	-18.6 (-25.5,-11.7)*	0.57	19.7	-18.6 (-25.0,-12.3)*	0.61	18.2^#^
**EPI-Q group**						
Low	-11.8 (-18.4,-4.2)*	0.41	13.9	-13.4 (-19.7,-6.6)*	0.50	12.7^#^
High	-26.8 (-38.3,-14.5)*^A^	0.80	23.6^#^	-24.8 (-35.6,-13.2)*	0.81	22.3^#^
**RSES group**						
Low	-21.8 (-35.0,-8.6)*	0.56	22.0	-20.2 (-32.8,-7.5)*	0.55	21.2
High	-16.6 (-24.8,-8.5)*	0.64	17.9	-17.7 (-24.9,-10.5)*	0.74	16.0^#^

* Denotes significant change in OHIP-49 score (P < 0.05), ^A^ denotes significant difference between high and low EPI-Q and RSES group respectively (P < 0.05), and ^#^ denotes clinical significance (change exceeds SEM).

### Multivariate analyses

The results from the multivariate analyses are shown in Table 3. It is seen that the relative increase in the explanation percentage by adding the EPI-Q score and RSES score to the controlling variables is 50% and 148% respectively of the variance in the OHIP-49 change score and 164% when adding both. Further the parameter estimates indicates that neither the RSES nor the EPI-Q score significantly explained the change in OHIP-49 score, whereas the high EPI-Q group was associated with a significantly higher improvement in OHIP-49 score compared to the low EPI-Q group.

**Table 3:** Multivariate analyses

**Explanation percentages of OHIP-49 change score**		
**Model 1.** Controlling variables	4.2%	
**Model 2.** Controlling variables + EPI-Q score	6.3%	
**Model 3.** Controlling variables + RSES score	10.4%	
**Model 4.** Controlling variables + EPI-Q and RSES scores	11.1%	
**Model 5.** Controlling variables + EPI-Q and RSES groups	15.8%	
**Parameter estimates**		
**Model 4**	**Estimate**	***p***
RSES score	1.0	0.09
EPI-Q score	-0.9	0.52
**Model 5**		
RSES group (low vs. high)	-2.1	0.78
EPI-Q group (high vs. low)	-20.6	0.01

## Discussion

This study is the first to investigate if and how NA and self-esteem influences the patient-reported effect of oral rehabilitation. Before concluding on the results, the limitations of the study must, however, be discussed. The population size was recognized as a limitation of the study; even though the study population was large enough to produce significant and reliable results for the entire population, differences between subgroups of participants were harder to find. Further, the population was selected from a dental school and the results should therefore be generalized cautiously to the entire population. Another limitation of the study regards the controlling variables. They were selected as they were thought to influence the OHIP-49 score in the study population, and even though the multivariate analyses showed that the controlling variables indeed explained some of the variance in OHIP-49 scores, it was recognized that the controlling variables in this study does not encompass all OHRQoL aspects, and other dental status variables, socio-economic status, and dental attendance could account for additional variance in the OHIP-49 score [8].

Even though it has been said that failure to adjust the baseline score for the RTM may lead to erroneously conclusions [24], no OHRQoL studies have so far done a statistically adjustment for RTM. By adjusting the pre-treatment OHIP-49 score, more valid results were expected and the procedure therefore strengthened the study. As a result of the adjustment, the ES increased and the SEM decreased in this study, indicating that adjustment for the RTM increased the significance of the change scores. The change in ES and SEM was, however, detected in all subgroups indicating that the adjustment did not have any impact on the influence of the personality traits on the change scores. For the findings of this study to be comparable to other studies, results on non-adjusted scores were also presented.

Statistical significant differences in OHRQoL between groups do not provide information on the meaningfulness of the difference neither from a clinical or patient perspective [38]. The clinical meaning was therefore prioritized and evaluated in this study. The MID, ES, and SEM were thought to complement each other well; the MID was generated by an anchor based method, whereas ES and SEM are distribution based methods. Further, the ES has been widely used but has some limitations regarding the sample heterogeneity whereas the SEM is more stable across samples [24]. The SEM was interpreted as clinical significance, and even though a more conservative approach using a cut-off value of 1.96 SEM has been applied to define clinical significance [39], a change exceeding 1 SEM was in this study considered sufficient as recommended in other studies [36,40].

In this study, treatment with RDP improved the OHRQoL statistically significant for the entire population, but did not improve the OHRQoL to the same level for all subgroups: a statistical and clinical significant higher post-treatment OHIP-49 score was found in the high EPI-Q and low RSES group compared to the low EPI-Q and high RSES group. In spite of the adjustment for the RTM, the difference in post-treatment scores was probably due to the high influence of the personality traits on the pre-treatment scores, i.e. the effect as measured by the change scores was not limited by high NA and low self-esteem. In fact, even though not significant, the linear association between the EPI-Q and RSES scores and OHIP-49 change score, indicated that participants with higher NA and lower self-esteem improved more in OHRQoL than participants with lower NA and higher self-esteem. On group basis this effect was even more pronounced; the high EPI-Q group improved the most in OHRQoL, and the magnitude of the change was large and exceeded the SEM indicating a clinically meaningful change. This somewhat unexpected effect is also seen in the multivariate analyses, which showed that even though the explanatory capacity of the EPI-Q score was limited, the influence when the participants were divided into groups were significant; the high EPI-Q group had a higher improvement in OHRQoL than the low group. The explanation for the conflicting results between the influence of the EPI-Q score and the EPI-Q groups on the change in OHRQoL is probably that the relationship between the EPI-Q and OHIP-49 change score was not a linear one. The model including the group variables simply seems to be a better fit to the data, indicating a jump in the improvement in OHRQoL around the mean EPI-Q score. The explanation percentages from the multivariate analyses were, however, in general low suggesting that no variable influenced the effect to a large degree.

Only two prospective intervention studies investigating the influence of NA on treatment effect is found in the literature. Both studies dealt with patients treated for coronary heart diseases and even though they did not investigate the influence of NA on the change in quality of life, both studies concluded that persons with high NA exhibited a relationship to reduced quality of life both before and after treatment [19,20]. This is in line with the results from this study; even though participants with high NA improved more than participants with low NA, the post-treatment OHIP-49 score in participants with high NA did not reach the level of the participants with low NA. Other studies concerning NA or self-esteem and OHRQoL do unfortunately not investigate if the personality traits influence the effect of treatment. Studies on self-esteem investigate if self-esteem is affected by oral treatments [41,42], and studies on NA have primarily been population or cross-sectional studies [8,10].

In the clinic, it is necessary to determine the indication for and putative effect of oral rehabilitation. The results from this study could assist clinicians in doing this when treatment with RDP is planned. The association of worse NA and self-esteem to worse OHRQoL both pre- and post-treatment should supposedly be addressed in the initial phase of the treatment planning and incorporated in the decision making. It is important, however, to know that not all hope is out for patients with high NA and low-self-esteem; it seems that in spite of the influence of the personality traits, treatment with RDP is not contraindicated in these patients. Whether the participants with high NA and low self-esteem are using their RDP more or less than the participants with low NA and high self-esteem is not known, but would be a highly interesting and relevant future investigation.

## Conclusion

Treatment with RDP improves the OHRQoL regardless of level of NA and self-esteem. High NA is associated with a large effect, but both high NA and low self-esteem is associated with poorer OHRQoL both before and after treatment.

## Competing interests

The author declares no competing interests.

## Author’s contributions

The author performed all steps of the study from conception through data collection and analysis and writing the paper.
